# Coding and Non-Coding Transcriptomic Landscape of Aortic Complications in Marfan Syndrome

**DOI:** 10.3390/ijms25137367

**Published:** 2024-07-05

**Authors:** Nathasha Samali Udugampolage, Svetlana Frolova, Jacopo Taurino, Alessandro Pini, Fabio Martelli, Christine Voellenkle

**Affiliations:** 1 Cardiovascular-Genetic Center, IRCCS Policlinico San Donato, 20097 Milan, Italy; nathasha.udugampolage@grupposandonato.it (N.S.U.); jacopo.taurino@grupposandonato.it (J.T.); alessandro.pini@grupposandonato.it (A.P.); 2 Molecular Cardiology Laboratory, IRCCS Policlinico San Donato, 20097 Milan, Italy; svetlana.frolova@grupposandonato.it (S.F.); christine.voellenkle@grupposandonato.it (C.V.); 3 Department of Biosciences, University of Milan, 20122 Milan, Italy

**Keywords:** Marfan syndrome, thoracic aortic aneurysm, heritable aortic aneurysms and dissection, clinical guideline, protein-coding and non-coding RNAs, biomarker, transcriptome, single-cell RNA-seq, epigenome, chromatin changes

## Abstract

Marfan syndrome (MFS) is a rare congenital disorder of the connective tissue, leading to thoracic aortic aneurysms (TAA) and dissection, among other complications. Currently, the most efficient strategy to prevent life-threatening dissection is preventive surgery. Periodic imaging applying complex techniques is required to monitor TAA progression and to guide the timing of surgical intervention. Thus, there is an acute demand for non-invasive biomarkers for diagnosis and prognosis, as well as for innovative therapeutic targets of MFS. Unraveling the intricate pathomolecular mechanisms underlying the syndrome is vital to address these needs. High-throughput platforms are particularly well-suited for this purpose, as they enable the integration of different datasets, such as transcriptomic and epigenetic profiles. In this narrative review, we summarize relevant studies investigating changes in both the coding and non-coding transcriptome and epigenome in MFS-induced TAA. The collective findings highlight the implicated pathways, such as TGF-β signaling, extracellular matrix structure, inflammation, and mitochondrial dysfunction. Potential candidates as biomarkers, such as miR-200c, as well as therapeutic targets emerged, like Tfam, associated with mitochondrial respiration, or miR-632, stimulating endothelial-to-mesenchymal transition. While these discoveries are promising, rigorous and extensive validation in large patient cohorts is indispensable to confirm their clinical relevance and therapeutic potential.

## 1. Background

Aneurysms of the thoracic aorta (TAA) are a consequence of the necrosis or degeneration of the cystic layer of the arterial wall, which is characterized by a decrease in smooth muscle cells (SMC), breakdown of elastin fibers, and an increase in the deposition of proteoglycans in the tunica media of the aortic wall [1]. TAA are often defined by the location and degree of aortic involvement: most TAA arise in the aortic root or ascending aorta (60%), followed by descending aorta (40%), aortic arch and, lastly, thoracoabdominal aorta [2].

Although the majority of TAA have degenerative etiology and the concurrent presence of cardiovascular risk factors, about 5% of TAA, especially in the ascending aorta, are linked to genetic conditions affecting connective tissue and familial predisposition [2]. In this regard, up to 20% of those who are typically affected have a first-degree relative who has a dilated thoracic aorta [1,3,4]. In the presence of familial patterns, clinical guidelines identify TAA based on the presence or absence of syndromic aortic aneurysm conditions, such as Marfan syndrome (MFS, OMIM #154700), Loeys–Dietz syndrome (LDS, OMIM #609192), or vascular Ehlers–Danlos syndrome (vEDS, OMIM #130050). For a better understating of the genetic involvement in syndromic TAA, mutations in the gene that encodes the Fibrillin-1 protein (FBN1) are mainly associated with MFS. A different group of genes encoding the transforming growth factor beta (TGF-β) signaling pathway (TGF-β receptors 1 and 2 (TGFBR1, TGFBR2), TGFB2, TGFB3, similar to Mothers Against Decapentaplegic 2 and 3 (SMAD2, SMAD3) and Sloan–Kettering Institute Proto-Oncogene (SKI)) give rise to TGF-β-related vascular disorders, which are linked to LDS (especially TGFBR1 or TGFBR2 mutations), vEDS (mutations in collagen type III alpha 1 chain gene (COL3A1)) and predispose patients to aggressive and widespread vascular conditions [5]. Predominant phenotypes related to connective tissue disorders (CTDs) encompass manifestations in the skin, musculoskeletal, visual, craniofacial, and cardiovascular systems [6], occasionally accompanied by functional impairment. Research revealed that about 30% of families affected by heritable thoracic aortic diseases (HTAD), without a confirmed diagnosis of MFS or a similar syndrome, carry a responsible, disease-causing genetic mutation within the recognized HTAD-related genes (for example, Actin Alpha 2 (ACTA2), myosin heavy chain 11 (MYH11), myosin light chain kinase (MYLK), notch receptor 1 (NOTCH1) and similar, as reported by Milewicz et al.) [7,8]. With the widespread adoption of whole exome sequencing among TAA patients and their relatives, new pathological and potentially significant genetic variations are being identified on a regular basis [8].

In nonsyndromic TAA, abnormalities are limited to the cardiovascular system. The majority of these conditions show autosomal dominant inheritance, but affected individuals do not exhibit external features of CTD or any other recurrent phenotype as in syndromic TAA [6]. Conditions that are usually considered nonsyndromic include familial thoracic aneurysms and dissections (also known as familial TAA), as well as TAA with bicuspid aortic valve (BAV) [6]. In contrast to abdominal aortic aneurysms, there is no general population screening protocol for TAA [1]. The identification of TAA commonly occurs as an unintended discovery and, to a lesser degree, through systematic examination [9]. Different sets of symptoms may arise depending on the size and location of the TAA. Over 95% of TAA do not show symptoms until they reach the point of dissection or rupture [10]. Nevertheless, guidelines suggest screening close family members of individuals with a history of TAA or aortic dissection. In cases where there is substantial evidence of a familial TAA, considering screening second-degree relatives is justified. It may also be reasonable to screen for TAA in patients whose first-degree relatives have experienced unexplained sudden cardiac death [11,12]. Regardless of its size, the onset of symptoms linked to TAA should lead to an immediate assessment for surgical intervention.

Due to the high occurrence of aneurysms in locations beyond the thoracic aorta among patients with familial TAA, computed tomography angiography (CTA) or magnetic resonance imaging (MRI) are recommended for entire morphological evaluation aorta [1]. A transthoracic echocardiogram (TTE) should be performed to check for valve-related conditions. It is advisable, according to current guidelines, to begin screening with imaging around the age of 25 or 10 years before the earliest case in a family group [1,13]. If the initial screening results are normal, follow-up imaging should be done at 5-year intervals. Instrumental diagnosis with TTE is the method of choice for assessing and following up on the diameters of the aortic root, ascending aorta, aortic arch, and descending aorta [1].

Therefore, periodic imaging of TAA should be performed to determine the necessity and timing of prophylactic surgery. To date, preemptive surgical correction of TAA is the most efficient approach to prevent aortic rupture and dissection [14,15], proving successful repair of TAA and long-term survival rates when compared to controls of similar age and gender. Recommendations outlined in guidelines regarding the criteria for considering proactive intervention are founded on the current knowledge of TAAs’ natural progression and the associated surgical risks [14].

**Figure 1:**
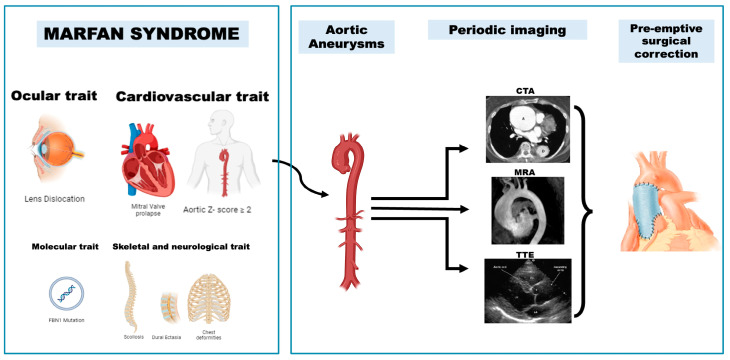
**Left** side: Multisystemic phenotype of MFS; **Right** side: diagnostic techniques recommended for MFS focus on the use of transthoracic echocardiography (TTE), computed tomography angiography (CTA), and magnetic resonance imaging (MRI). Image created using Biorender.com.

Aside from prophylactic surgery, medical treatment has been reported to slow the rate of aortic enlargement [1,14]. Recommendations for syndromic patients, especially in MFS, include beta blockers, which have proved efficacy in slowing the expansion of TAA. Current guidelines advocate for the administration of beta blockers to all MFS patients to reduce the rate of aortic enlargement [14]. Despite their specific indication, beta blockers are frequently prescribed to TAA patients without MFS (Figure 1). More recently, Angiotensin II receptor blockers (ARB) have been found to reduce the progression of aortic dilation in MFS patients. This discovery has prompted guideline recommendations for ARB in all MFS patients and preclinical data suggest potential benefits for LDS, likewise [14]. However, similar to beta blockers, there is limited evidence supporting the efficacy of ARB in patients with TAA who do not have MFS or LDS [1].

To date, gold standard diagnostic techniques for MFS focus on the use of TTE, CT scan, and MRI [9]. Biomarkers allowing early detection of disease progression in TAA would represent an important advance for the prognosis and management of the disease.

## 2. Methods

For the narrative review, literature research was conducted by employing the medical databases “PubMed” and “Scopus” as well as the search engine “Google Scholar”. The following keywords were applied: “thoracic aorta aneurysm”, “TAA”; “Marfan syndrome”, “MFS”; “transcriptomic”; “RNAseq”, “single-cell RNAseq”; “microarray”, “high-throughput”; “epigenetic”; “non-coding RNA”; “miRNA”; “lnRNA”; “biomarker”. All these search terms were also used in combination with the Boolean operators AND and OR, along with other diseases described in this review: “Loeys–Dietz syndrome” or “LDS”, “vascular Ehlers–Danlos syndrome” or “vEDS”, “bicuspid aortic valve” or “BAV”. We considered relevant publications from 2007 until May 2024. Other articles were selected from the reference list of the retrieved publications.

## 3. Genome-Wide Gene Expression Analysis of MFS

**Figure 2:**
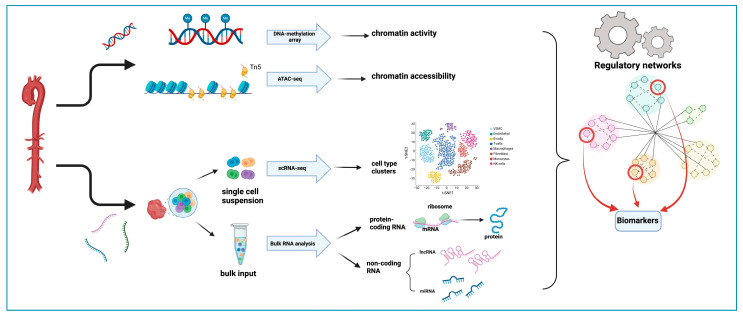
Genome-Wide Gene Expression and Epigenetic Analysis of MFS. Image created using Biorender.com.

The unmet need for non-invasive biomarkers for both the diagnosis and the prognosis of complex diseases such as MFS underscores the utility of high-throughput platforms. These techniques permit a simultaneous analysis of various aspects of a disease. For instance, a seroproteomic approach enables the concurrent measurement of a multitude of cytokines and chemokines, while an epigenetic approach facilitates the identification of chromatin modifications associated with pathological conditions. Particularly promising is the transcriptomic approach, exploiting the entire transcriptome, constituted by both coding (messenger RNAs, mRNAs) and non-coding RNAs (ncRNAs), such as microRNAs (miRNAs), long non-coding RNAs (lncRNAs), and circular RNAs (circRNAs). Pioneering transcriptome studies of aortic aneurysms from MFS patients have demonstrated the potential to identify differentially expressed mRNAs, miRNAs, and lncRNAs that may play crucial roles in the pathogenic mechanisms of the disease (Figure 2).

### 3.1. Transcriptomic Studies of Coding Transcripts in MFS

In 2007, Yao et al. conducted one of the first genome-wide expression analyses investigating MFS [16]. Employing microarray analysis, they identified more than 260 differently expressed genes in skin fibroblasts of MFS patients compared to healthy controls. Notably, modulation of known regulators of the TGF-β pathway was found, such as reduced levels of the repressor vitamin D3 receptor (VDR), together with an increase in the activator tuberous sclerosis 2 (TSC2). Moreover, downstream targets of TGF-β showed expression changes, including the cell adhesion regulator LIM domain only 7 (LMO7), matrix metalloproteases (ADAM metallopeptidase domain 12 (ADAM12)), matrix metallopeptidase 1 (MMP1), TIMP metalloproteinase inhibitor 3 (TIMP3) and collagens (COL3A1, collagen type I alpha 2 chain (COL1A2)). The observed alterations are consistent with enhanced TGF-β activity, suggesting a potential key role of this pathway in TAA development in MFS patients. Subsequent research in human aortic tissues has supported these first findings, confirming the pathway’s contribution to the disease [17,18].

An exploratory gene expression analysis was conducted using microarray in fibrillin-1 hypomorphic mice (mgR/mgR), characterized as a reliable model of severe MFS. Interestingly, out of 159 genes deregulated in the ascending aorta of mgR/mgR mice, 140 were upregulated in the MFS mouse compared to the WT [19]. Gene ontology analysis showed an overrepresentation of terms related to immune functions. Furthermore, for several relevant genes, the observed modulations were validated by PCR, such as C-C motif chemokine ligand 2 and 5 (Ccl2, Ccl5), C-X-C motif chemokine receptor (Cxcr1), and interleukin 1 beta (IL1β). Although this study did not conduct further investigations, the validity of this dataset is corroborated by similar findings in recent studies [20,21].

The transcriptome of mgR/mgR mice was further analyzed by employing high throughput, massively-parallel sequencing (RNA-seq) [20]. Investigation of the aortic tissue of mgR/mgR mice uncovered 248 differentially expressed genes. Gene ontology analysis identified associations relevant to cardiovascular complications and MFS. PCR and immunoblot validation confirmed the observed modulations for several genes, including the induction of insulin-like growth factor binding protein 2 (Igfbp2) and Ccl8, alongside the down-modulation of microfibril-associated protein 4 (Mfap4). Additionally, protein expression of pSmad2 and extracellular signal-regulated kinase 1/2 (pErk1/2) was induced. It has been shown that Igfbp2 promotes ERK1/2 signaling [22], which, in turn, contributes to load-induced cardiomyopathy in Marfan mice [23]. These observations prompt speculations about a possible role of Igfbp2 regulating pERK1/2 levels in mgR/mgR mice. Furthermore, Mfap4 interacts directly with FBN-1 and is crucial for microfibril development and maintenance of extracellular matrix (ECM) proteins [24]. Together with the strong induction observed in mgR/mgR mice by Bhushan, these findings point to Mfap4 as a potential regulator in MFS. This study also sheds light on a possible involvement of secreted phosphoprotein 1 (Spp1) in MFS, known for its role in chronic inflammation [25] and proposed as a biomarker for CVD [26,27]. Additionally, several chemokines were found to be highly up-modulated, suggesting a complex role of inflammation in the aorta of mgR/mgR mice.

A subsequent comprehensive study combining transcriptomics and metabolic analysis in the aortas of Fbn1^C1039G/+^ mice aimed to discover novel molecular mechanisms underlying TAA formation [28]. This mouse model is heterozygous for the most common class of mutation causing MFS, resulting in progressive aortic root dilatation [29]. RNA-seq analysis revealed reduced expression of genes related to fatty acid β-oxidation, as well as of most mitochondrial complex subunits, including mitochondria- and nuclear-encoded genes, mitochondrial biogenesis, and function. Among these, there was the mitochondrial transcription factor A (Tfam), controlling the transcription, replication, and stability of mitochondrial DNA (mtDNA) [30]. In contrast, genes involved in mitochondrial uncoupling (Ucp2) and glycolytic rewiring (hypoxia-inducible factor 1 subunit alpha (Hif1a), myelocytomatosis proto-oncogene (Myc) were upregulated.

Silencing of Fbn1 in primary murine VSMCs confirmed reduced Tfam levels and mtDNA content, resulted in decreased mitochondrial respiration and increased lactate production, and displayed senescence and inflammation features. Intriguingly, these results were confirmed also in TAA tissue and primary skin fibroblasts derived from MFS patients. Moreover, mitochondrial dysfunction was observed seeding healthy VSMCs in ECM matrices produced by Fbn1-deficient VSMCs, bringing further evidence that the mitochondrial function of VSMCs is controlled by the extracellular matrix. To investigate the importance of mitochondrial dysfunction in the development of aortic aneurysm, a conditional mouse model was generated, displaying VSMC-specific mitochondrial dysfunction by depleting Tfam. In fact, these mice lost vascular contractile capacity and developed aortic aneurysms and lethal dissections, corroborating the suggested relationship. Then, consequences of enhanced mitochondrial function were investigated, treating Fbn1^C1039G/+^ mice with an NAD+ precursor, boosting mitochondrial metabolism by increasing peroxisome proliferator-activated receptor gamma coactivator 1-alpha (Pgc1-α) and Tfam expression [31,32]. Indeed, this treatment rapidly reverted aortic aneurysm by restoring mitochondrial metabolism in Fbn1^C1039G/+^ mice. Although the findings of this extensive study were discovered in an MFS mouse, intriguingly, they could also be confirmed in the patient context. Thus, targeting the mitochondrial metabolism could represent a very promising therapeutic strategy for treatment in MFS patients. Furthermore, considering that the observed mitochondrial decline appears at the onset of aortic disease in Fbn1^C1039G/+^ mice, involved key players could represent potential biomarkers for the progression of the disease.

Verhagen et al. performed a multi-omics approach using aortic tissue from patients with two groups of MFS variant types (haplo insufficient HI, dominant negative DN) to determine whether the molecular mechanisms are affected by these variations [33]. More than 1500 genes were modulated, showing a strong overrepresentation of inflammatory and mitochondrial pathways in all MFS patients compared to healthy donors, corroborating the studies performed in mouse models. Proteomic analysis identified nearly 500 deregulated proteins, and the most significant pathways were related to mitochondrial dysfunction and oxidative phosphorylation, corroborating the findings by RNA-seq. Moreover, bioinformatic analysis of the datasets predicted the repression of genes associated with mitochondrial respiration and metabolism. Accordingly, the comparison of cultured VSMCs from MFS patients and controls confirmed decreased mitochondrial respiration, reaching significance only in HI variants. Still, due to the small sample number, the effect of DN variants cannot be excluded. Noteworthy, no distinctive TGF-β signature was found in MFS patients, neither on gene nor on protein expression levels. This might be due to the advanced state of the pathology, considering the suggested dual role of TGF-β signaling in aneurysm progression [34,35,36]. The altered mitochondrial function was observed in MFS mice and patients, also by Oller et al. [28], corroborating the results. Collectively, the findings of these two studies emphasize the potential of mitochondrial pathway members to function as markers for TAA progression or as therapeutic targets for MFS treatment.

### 3.2. Transcriptomic Studies of Non-Coding Transcripts in MFS

While less than 2% of the human genome is transcribed into protein-coding RNAs (mRNAs), the vast majority is transcribed into RNAs that lack protein-coding potential (ncRNAs) [37,38]. These are categorized based on their nucleotide (nt) length: small (<200 nt) and long (>200 nt) ncRNAs. MiRNAs, ∼22 nt long, regulate the expression of their target genes through sequence-specific recognition. First, the mature miRNA interacts with Argonaute proteins, forming the RNA-induced silencing complex (RISC) [39,40]. Subsequently, the RISC identifies and binds to the target mRNA, resulting in degradation and/or translational inhibition of the target. Each miRNA can have multiple targets and a single mRNA may have several miRNA recognition sequences, generating complex regulatory networks [41]. LncRNAs have different functions. Some nuclear lncRNAs can act at the epigenetic level, regulating transcription initiation by assembling transcriptional activators and repressors [42]. Others bind directly to mRNAs, thereby modulating the translation and/or stability of their target. LncRNAs can also function as competing endogenous RNAs, sequestering miRNAs [43,44] through binding, consequently mitigating the repression of the target mRNA [45]. Recent studies have demonstrated that the dysregulation of ncRNAs plays a role in multiple diseases, including CVD [46,47,48,49,50,51,52,53,54]. Since ncRNAs are released into the blood, show high stability, and are readily measurable, this RNA class, and in particular miRNAs, represents an enormous reservoir for biomarkers discovery for both diagnostic and prognostic applications [46,47,48,49,55,56].

Since miRNA and mRNAs form an interdependent regulatory network, parallel profiling of long and small RNA species is particularly informative. An integrated miRNA and mRNA expression profiling by microarrays in the peripheral blood of MFS patients identified 28 miRNAs and 32 mRNAs that were significantly altered in MFS [57]. Among validated candidates, inverse mRNA–miRNA correlations were found between 8 miRNAs and 5 mRNAs associated with vascular pathology, inflammation, and telomerase regulation. Moreover, several miRNAs showed a significant correlation to the clinical parameters of MFS patients. MiRNA-200c, linked to cardiovascular disease [58] before, correlated significantly with aortic root status (Z-score), while 7 other miRNAs correlated with left ventricular end-diastolic diameter. It is particularly noteworthy that no significant correlation was found between protein-coding RNAs and clinical parameters, which underscores the potential of non-coding RNA as biomarkers in MFS.

A pioneering study on lncRNA expression profiles in dilated aortic tissues of MFS patients was conducted in 2020 [59], using microarrays. Differential expression analysis identified significant changes in 294 lncRNAs and 644 mRNAs. Gene ontology analysis indicated cell adhesion, elastic fiber assembly, ECM organization, and inflammatory response. KEGG pathways associated with the signature included TNF signaling, focal adhesion, ECM receptor interaction, and mitogen-activated kinase-like protein (MAPK) signaling. When lncRNA–mRNA co-expression network analysis was performed, five lncRNA–mRNA pairs were further confirmed by PCR. Interestingly, the strongest induced lncRNA X inactive specific transcript (XIST) was linked to a tissue inhibitor of TIMP 4, playing a central role in the pathogenesis of MFS [60]. Further investigation of the co-expression networks identified in this study is crucial for validating their significance in MFS and assessing their potential as novel markers.

In the aforementioned study by Bhushan et al. [20], H19 was highly upregulated in the aortic tissue of mgR/mgR mice. Interestingly, this non-coding lncRNA is also induced in heart failure patients [61] and associated with an elevated risk of coronary artery disease [62]. Hence, it could represent another promising avenue for exploration as a potential disease predictor or therapeutic target in MFS.

D’Amico et al. performed an extensive study comparing aortic tissue from MFS and non-MFS TAA. Morphological, ultrastructural, and phenotypic analyses alongside miRNA and mRNA profiling were carried out [18]. Microscopic examination revealed increased loss and fragmentation of elastic fibers in the tunica media of MFS TAA. Ultrastructural investigations displayed a more severe ECM degeneration and morphological changes in SMC of MFS tissue. Immunohistochemistry showed increased angiogenic remodeling, elevated MMP-2 expression, inflammation, and SMC turnover in MFS TAA; interestingly, in vitro studies with SMCs confirmed these findings. Upon RNA profiling, 25 down-modulated miRNAs and a single up-modulated miRNA (miR-632) were identified in MFS TAA. KEGG pathway analysis associated these miRNAs with cell proliferation, ECM structure/function, and TGF-β-signaling [63]. Gene expression analysis revealed 35 modulated genes in MFS. Target prediction identified 11 miRNAs as potential regulators of 7 deregulated genes belonging to the cadherin 1/anaphase-promoting complex (CDH1/APC) and cyclin A2/tumor protein p53 (CCNA2/TP53) signaling pathways. This specific miRNA and mRNA signature was associated with increased angiogenic remodeling, likely contributing to early onset and more severe clinical outcomes of MFS. These findings contribute to a deeper understanding of the molecular mechanisms driving TAA development and progression in MFS. Moreover, this study provides a carefully investigated dataset, valuable for further exploration of potential MFS biomarkers. Indeed, the role of singularly up-modulated miRNA, miR-632, was further analyzed [17]. This miRNA was identified in cancer as an important epigenetic regulator of DnaJ heat shock protein family (Hsp40) member B6 (DNAJB6) [64], crucial for inhibiting the epithelial-to-mesenchymal transition via the Wnt/β catenin pathway [65,66]. It has been shown that TGF-β/Wnt-β catenin signaling can induce fibrosis [67] and endothelial-to-mesenchymal transition (End-Mt) [68], processes previously observed in aortic aneurysm [69]. In accordance, also in the investigated MFS TAA tissues, upregulation of miR-632 correlated with DNAJB6 inhibition and induced Wnt/β catenin signaling as well as End-Mt and fibrosis markers. Moreover, overexpression of miR-632 in non-MFS aorta fragments blocked DNAJB6, in turn stimulating Wnt/β catenin signaling, End-Mt, and fibrosis. Intriguingly, treatment with TGF-β, directly implicated in MFS, induced miR-632 upregulation with consequent activation of the aforementioned processes. These findings highlight the complexity of the involved pathways and encourage further investigation for key players within regulatory networks leading to TAA progression in MFS.

A recent, very detailed study explored whether and how specific FBN-1-regulated miRNAs mediate inflammatory cytokine expression and elastic laminae degradation in TAA of MFS [21]. Utilizing Fbn1^mgR/mgR^ mice, time-dependent miRNA expression profiling at early (4 weeks) and late (10 weeks) TAA stages was conducted. While at the initial stage, 17 miRNAs are deregulated, at the later stage, 129 miRNAs are differentially expressed. Both stages, early and late, exhibit signatures related to inflammatory responses and ECM-related pathways. Additionally, RNA-seq analysis with TAA tissues from MFS patients was performed and compared to the murine model, revealing upregulated pro-inflammatory cytokines and matrix metalloproteinase as common features. Notably, for miRNA-122, displaying the strongest down-modulation at 10 weeks, post-transcriptional induction of Ccl2, IL1β, and MMP12 was proved. Taking advantage of a milder murine MFS model (Fbn1^C1041G/+^) [70], 70-week-old tissues could be investigated, showing similar results for miRNA-122 and its target genes. This miRNA is predominant in the liver, constituting up to 70% of the total miRNA population in mice [71]. Knockout of miR-122 in mice caused increased inflammatory responses in the liver, including induction of Ccl2, IL6, and TNF-α [72]. This is consistent with in silico analysis predicting a significant role of miR-122 in the regulation of inflammatory pathways in the MFS TAA tissues. Numerous experiments showed that the reduced levels of miR-122 in the aortic wall of Fbn1^mgR/mgR^ mice play an important role in the connection between FBN-1 deficiency and hypoxia. Upstream of miR-122, a disruption of integrin signaling and upregulation of HIF1α was observed, while downstream targets Ccl2 and MMP12 were increased. Inhibition of HIF 1α by Digoxin treatment of Fbn1^mgR/mgR^ mice induced miR-122 levels, leading to reduced levels of Ccl2 and MMP12, resulting in diminished elastic laminae fragmentation and ameliorated aortic dilation. Based on extensive experimental evidence, this detailed study underscores the potential of miR-122 as a therapeutic target for MFS-induced TAA.

### 3.3. Single Cell Transcriptomic Studies in MFS

The human aorta, composed of various cell types, including VSMC, fibroblasts, endothelial cells (ECs), and immune cells, plays a pivotal role in maintaining aortic wall integrity. Aberrant SMC phenotype is a hallmark of aortic aneurysm and a primary focus of TAA research [73,74,75]. Single-cell RNA sequencing (scRNA-seq) allows for transcriptome-wide gene expression measurements at the resolution of individual cells. Usually, as a first step, a single-cell suspension is obtained through enzymatic cell dissociation, a challenging task given the human aorta’s particularly strong mechanical properties [76]. Single-cell isolation can then be achieved by either plate-based protocols, which involve sorting or manual deposition of cells into wells (e.g., Smart-seq) [77], or by droplet-based methods, capturing the individual cells in microfluidic droplets [78,79]. Whereas split-pool ligation-based transcriptome sequencing (SPLiT-seq) does not require cell separation but identifies the cellular origin of each RNA transcript through several cycles of combinatorial barcoding of permeabilized cells [80]. Among the most commonly used platforms is 10X Genomics Chromium. The individual cells are co-encapsulated with mRNA capture beads in droplets or microwells. Subsequently, cell barcodes are incorporated into cDNA from thousands of cells in parallel [81,82]. For each of these cells, expression profiles are produced by sequencing. All profiles are clustered based on their expression using an unsupervised method, and the resulting clusters are then associated with different cell types based on canonical markers [83].

Pedroza exploited the scRNA-seq technology to analyze aortic tissue from Fbn1^C1041G/+^ and healthy mice and a single MFS patient [84]. All major aortic cell types were identified, and top gene markers for each cluster were identified by differential gene expression and separated by genotype. A distinct, MFS-specific cluster was found, positioned by bioinformatic tools with SMCs and adjacent to fibroblasts, suggesting an SMCs subset undergoing phenotypic modulation toward a fibroblast-like state (“modSMCs”). Although comparison with the atherosclerosis mouse model (ApoE^−/−^) data indicated very similar patterns of SMC modulation, the overexpression observed for serpin family E member 1(Serpine1) and Kruppel-like transcription factor 4 (Klf4) was specific for MFS. Transcriptomic comparison between SMC and modSMC identified several biological processes to be activated by modulation of the phenotype, such as cell adhesion, proliferation, and collagen fibril organization. The absence of modSMCs in young mice and in non-dilated descending thoracic aortas of adult mice highlighted the temporally and spatially specificity of the modSMCs cluster. scRNAseq with aortic tissue from a single MFS patient showed a similar pattern of SMC phenotype modulation, with induced TGF-β-responsive genes associated with modSMCs in both human and murine datasets. These findings suggest enriched TGF-β signaling and Klf4 overexpression as potential upstream drivers of SMC phenotype change during aneurysm development in MFS, implicating Klf4 as a critical target for further studies.

However, a study by Dawson et al. examining TAA from three MFS patients revealed different findings [85]. ScRNA-seq provided a detailed characterization of SMC and fibroblast phenotypes in the human aortic wall. Expression changes of marker genes across fibroblasts, intermediate SMCs/fibrocytes, and SMCs indicate a spectrum of phenotypes rather than distinct subsets, consistent with previous findings in atherosclerosis [86,87] and MFS [84,88]. Aortic MFS tissues showed an increase in de-differentiated, proliferative SMCs with reduced differentiation and contractile markers (myocardin (MYOCD), MYH11), while ECM marker COL1A1/2 was induced in fibroblasts. Compared to earlier studies in MFS patients, COL1A1 deregulation is in accordance, while MYOCD and MYH11 showed opposite modulation. Although TGFB1 was induced in fibroblasts, TGF-β receptors and SMAD were reduced in SMCs, fibroblasts, and ECs. The observed decrease in canonical TGF-β signaling, in contrast to previous studies [84,88], emphasizes the intricate mechanisms driving changes within specific subpopulations of aortic cells. A recent study employing scRNA-seq was conducted on aortic tissues of Fbn1^mgR/mgR^ mice [89], which display a fully penetrant dissecting phenotype [35], mirroring closely the events occurring in MFS patients. Two discrete subpopulations of aortic cells (SMC3 and EC4) were identified to be MFS-specific. SMC3 is characterized by the induction of genes related to ECM formation and nitric oxide signaling, while the EC4 transcriptome is enriched in SMC, fibroblast, and immune cell-related genes. Bioinformatics analysis predicted close phenotypic modulation between SMC3 and EC4, consequently analyzed together as “MFSmod”. Close transcriptomic homology was predicted between the MFSmod and the modSMCs identified by Pedroza in mice and humans [84]. However, for the MFSmod transcriptome, no association with TGF-β signaling was observed, possibly due to the different mouse models, displaying dissecting (Fbn1^mgR/mgR^) and not dissecting aneurysm (Fbn1^C1041G/+^). Aligning with Dawson’s study [85], upregulation of TGF-β1 and TGF-β2 ligands and inhibitory Smad7 was associated with the SMC1 cluster. Losartan treatment of Fbn1^mgR/mgR^ mice, which inhibits angiotensin II type I receptor (At1r) signaling and mitigates TAA progression in MFS mice [90], resulted in the absence of MFSmod cells. This sophisticated study identified alterations in the identity of aortic cells uniquely associated with dissecting TAA and responsive to losartan-mediated mitigation MFS mice. Acknowledging the inherent limitations of mouse models, the observed effects of losartan treatment in MFS mice still offers promising prospects for further therapeutic interventions in patients.

## 4. Epigenetic Studies in MFS

Epigenetics is the study of stable and hereditary changes regulating gene expression without altering the DNA sequence, such as DNA methylation [91], histone modification [92], and post-transcriptional regulation of ncRNA [93,94]. Such changes can have a strong effect on the recruitment of transcription factors and on the downstream transcriptional machinery [95,96,97,98,99], with consequences for numerous biological processes and diseases, such as CVD [100,101]. Techniques to study epigenetic changes include the treatment of DNA with bisulfite. During this process, unmethylated cytosines are deaminated to uracil, while methylated cytosines remain unchanged. This allows for the distinction and quantification of methylated from unmethylated cytosines in genomic DNA, which can be achieved using either microarrays or deep sequencing. Another method for studying epigenetics is the Assay for Transposase Accessible Chromatin with high-throughput sequencing (ATAC-seq). It employs an engineered, hyperactive Tn5 transposase to cut open chromatin and insert sequencing adapters simultaneously. These tagged DNA fragments are then sequenced and mapped, providing information about chromatin accessibility at a genome-wide scale. This method can be combined with single-cell sequencing (Single-cell ATAC-seq).

In 2021, van Andel performed the first epigenome-wide association study (EWAS) with peripheral whole blood from nearly 200 MFS patients [102]. DNA-methylation arrays were utilized to investigate methylation of CpG-sites across the entire genome. Among differentially methylated positions (DMP), 28 showed significant correlation with aortic diameters in patients, and 7 of these were associated with CVD-relevant genes (histone deacetylase 4 (HDAC4), insulin-like growth factor 2 mRNA binding protein 3 (IGF2BP3), castor zinc finger 1 (CASZ1), sidekick cell adhesion molecule 1 (SDK1), protocadherin gamma subfamily A (PCDHGA1), iodothyronine deiodinase 3 (DIO3), and protein tyrosine phosphatase receptor type N2 (PTPRN2)). Moreover, several DMPs were significantly correlated with changes in the aortic diameter or with clinical events. Further investigation of differentially methylated regions uncovered a gene cluster of protocadherins on chromosome 5, not reported before in MFS. This cluster includes genes related to cell adhesion, suggesting potential functional involvement. However, their specific relevance is yet to be determined. Interestingly, no methylation loci in the FBN1 gene vicinity are associated with the MFS phenotype. Noteworthy, no significant correlations were observed with non-cardiovascular phenotypic features of MFS, indicating specificity of the identified DMPs. This study offers novel insights into the epigenetic architecture of MFS. However, to confirm and extend these findings, it is essential to evaluate the functional relevance of the identified loci and replicate the study in other MFS populations.

Although the pathological consequences of the FBN1 mutation are systematic, aneurysms observed in MFS patients are usually located in the aortic root, while adjacent ascending aorta and arch remain unchanged. Researchers hypothesized that this difference is due to different embryological origins, specifically the cardiac second heart field (SHF) and neural crest [103]. To investigate these complex mechanisms, a SHF lineage-traced Fbn1^C1041G/+^ strain was generated, followed by integrated multi-omics analysis (scRNA-seq and ATAC-seq), stratified by embryological origin. SMC phenotypic modulation associated with MFS aneurysm was identified in both SHF and neural crest-derived cells. However, transcriptomic responses showed differences between lineages. SMCs with SHF origin overexpressed collagen synthetic genes and small leucine-rich proteoglycans, while SMCs with neural crest origin activated chondrogenic genes. These findings suggest that overactivation of the collagen synthetic pathway in SHF-derived SMC may contribute to the collagen depositions in the aortic root of MFS patients [88], thereby promoting focal aortic root aneurysm formation. Integrated scRNA-seq and ATAC-seq analysis identified the enrichment of Twist1 and Smad2/3/4 complex binding motifs in SHF-derived modulated SMC. These findings are particularly intriguing since activation of the TGF-β-Smad2/3/4 signaling axis is known to participate in aneurysm progression in murine MFS models [35,104]. Moreover, transcription factor twist family basic helix-loop-helix transcription factor 1 (TWIST1) has been implicated in pathological fibrosis [105], the silencing of SMC contractile genes [106], and the modulation of the SMC phenotype in coronary artery disease [107]. TWIST1 overexpression caused an increase in collagen and SLRP in vitro, suggesting a TWIST1-driven increase in collagen synthesis in SHF-derived SMC in MFS aneurysm. Collectively, the integrated multi-omics approach revealed that the embryologic origin influences the transcriptional response of SMC phenotype modulation in TAA of MFS. Additionally, transcription factor TWIST1 was discovered as a promising target for further mechanistic investigation.

## 5. Genome-Wide Gene Expression and Epigenetic Analysis in Congenital, Non-MFS TAA

Comparing studies with other congenital TAA can offer valuable insights. Firstly, it aids in identifying unique gene expression or methylation patterns specific to MFS, shedding light on the underlying molecular mechanisms of MFS pathogenesis. Secondly, it may unveil common molecular pathways and genetic signatures contributing to aneurysm formation and progression across different forms of congenital TAA.

### 5.1. Congenital, Syndromic (vEDS, LDS)

The comparison of genome-wide findings between MFS and vEDS or LDS is challenging due to the scarcity of related studies on these latter conditions. This may be explained by their lower prevalence rates. While MFS occurs in 1–5 per 10.000 individuals, vEDS occurs in only 1–9 per 100.000. According to ORPHANET, the prevalence of LDS remains unknown, which could be attributed to its recent identification (first described in 2005), together with the diagnosis complexity [108].

vEDS is caused by autosomal, heterozygous mutations in COL3A1, encoding the pro-α 1 chain of collagen III, a major structural matrix element in blood vessels and hollow organs [109]. It is thought that the reduced amounts of collagen III cause a loss of structural integrity within the extracellular matrix, leading to manifestations of vEDS [110,111,112]. These include arterial dissection, the main risk of premature death in vEDS patients, that, unlike in MFS or LDS, often occurs spontaneously, without prior aneurysm [104,113,114,115]. For the discovery of disease-relevant pathways, Bowen et al. generated two different mouse models (Col3a1^G209S/+^ and Col3a1^G938D/+^) carrying heterozygous mutations in Col3a1 previously found in vEDS patients [116]. Both the severe Col3a1^G938D/+^ and mild Col3a1^G209S/+^ murine models displayed spontaneous vascular rupture and death, thus recapitulating the vEDS phenotype. RNA-seq with aortic tissue showed excessive PLC/IP3/PKC/ERK signaling in vEDS mice, suggesting members of this pathway as major mediators of vascular pathology. Intriguingly, in MFS mouse models, beneficial effects from inhibition of the same signaling pathway had been demonstrated previously [117]. Indeed, treatment with pharmacological inhibitors of either ERK1/2 or PKCβ could prevent death due to aortic rupture also in Col3a1^G938D/+^. Instead, while the reduction in blood pressure by drug treatment mitigates TAA in MFS and LDS mice [29,89,104,115,116,117,118,119], in Col3a1^G938D/+^ mice, drug-reduced blood pressure did not improve survival. In vEDS patients, an increased vascular risk associated with pregnancy has been observed [120]. In MFS mice, Bowen et al. demonstrated previously that oxytocin release during lactation induces PLC/IP3/PKC/ERK signaling, causing aortic dissection [121]. Similarly, also in Col3a1^G209S/+^, inhibition of oxytocin signaling led to decreased ERK activation and downregulation of ERK target gene expression, resulting in a reduced risk of rupture. In adolescent young men with vEDS, an enhanced risk of vascular dissection has been observed, emerging toward the end of puberty [112,120]. In Col3a1^G938D/+^ mice, treatment with androgen receptor antagonists could rescue this elevated risk at sexual maturity and was correlated with changes in ERK1/2 activation in the aortic wall. The androgen signaling pathway might play a role also in MFS, where the occurrence and severity of TAA in male patients is higher compared with females [122]. This comprehensive study contributes to a deeper understanding of vEDS pathogenesis, identifying the PLC/IP3/PKC/ERK axis as a promising targetable pathway. Moreover, these vEDS mouse models could be a valuable tool for the discovery of biomarkers of disease risk and progression.

Recent RNA- and miRNA-seq studies with dermal fibroblasts derived from vEDS patients identified relevant biological networks [123]. MiRNAs deregulation was associated with critical cellular functions involved in vEDS pathophysiology, such as autophagy, proteostasis, and mTOR signaling [124]. Regulatory network analyses revealed interactions among miRNAs, lncRNAs, and candidate target genes. A total of 52 lncRNAs potentially acting as sponges for miR29a-3p and miR-29b-3p were identified, with NEAT1 being the most significantly downregulated ncRNA, known to function as a molecular sponge for both miRNAs [125]. Predicted targets of both miRNAs are linked to extracellular matrix organization and autophagy–lysosome pathway [126]. miRNA-29b, also induced in the aortic tissue of Fbn1^C1039G/+^ MFS mice, has been implicated in early aneurysm development [60], suggesting an analogue function in vEDS. Unravelling the miRNA–mRNA–lncRNA interactions of complex diseases might be particularly useful for identifying suited biomarkers and therapeutic targets.

Yu et al. investigated peripheral blood circulating endothelial cells derived from LDS patients, discovering overexpression of lncRNA AK056155, further confirmed in patients’ aortic aneurysm tissues [127]. Functional studies showed that AK056155 expression was enhanced by TGF-β1 and reduced by inhibitors of phosphatidylinositol 3-kinase (PI3K) or AKT serine/threonine kinase (AKT). This involvement of AK056155 in LDS through the PI3K/AKT pathway suggests its potential as a biomarker candidate for further studies. Interestingly, scRNA-seq in the aortic tissue of MFS patients identified PIK3/AKT as a key pathway in the fibroblast clusters of MFS [85].

### 5.2. Congenital, Nonsyndromic (BAV)

An early study on epigenetic changes in TAA formation in MFS and BAV, using patient-derived VSMC [128], revealed complex TGF-β1 independent dysregulation of Smad2 signaling for both TAAs. This dysregulation was characterized by an SMC-specific, heritable activation and overexpression of Smad2, associated with histone H3 modifications near the transcription start site (TSS) on the Smad2 promoter. Although no MFS or BAV-specific epigenetic pattern was found, this study gave the first evidence of epigenetic regulation involvement in TAA development.

Genome-wide DNA methylation and gene expression analysis with TAA tissues from BAV and tricuspid aortic valve (TAV) patients were also performed [129]. A total of 27 CpG probes in 9 genes showed significant differential methylation and specific expression for BAV. The strongest differences in both methylation and expression levels were observed in protein tyrosine phosphatase non-receptor type 22 (PTPN22), which exhibited hypermethylation and decreased expression in BAV relative to TAV tissues. This protein tyrosine phosphatase is known as a key regulator of T cell signaling, associated with numerous autoimmune diseases, such as type 1 diabetes, rheumatoid arthritis, and Graves’ disease [130]. Decreased PTPN22 expression may contribute to increased T cell activity, which is associated with increased SMC apoptosis and ECM degradation [131], leading to earlier and more frequent ascending aneurysm formation and also associated with MFS [132]. The discovery of epigenetic regulation of PTPN22 expression could provide insights into the underlying mechanisms of aneurysm development in individuals with BAV. Several genes implicated in cardiovascular development exhibited differential methylation but showed no differences in expression. Among these, ACTA2 is involved in the syndrome of familial thoracic aortic aneurysms and dissections when mutated [133] or GATA-binding protein 4 (GATA4), which plays a crucial role in myocardial differentiation, function, and embryogenesis [134]. It is conceivable that changes in methylation patterns of genes associated with cardiovascular development could contribute to the development of aneurysms in BAV patients.

In summary, comparisons with other congenital, syndromic, and nonsyndromic conditions that induce TAA and pose a risk of deterioration to dissection suggest common features, such as TGF-β pathway or ERK pathways. To discover reliable biomarkers for progression, highly specific for each of the described conditions, studies on every level are necessary. Further investigation through epigenetic and transcriptomic analyses, including single-cell level studies, is imperative to unravel the intricate regulatory networks underlying TAA development. Integrating data from these diverse approaches will provide a comprehensive understanding of the molecular mechanisms driving TAA pathogenesis.

## 6. Conclusions

Currently, reliable predictors of the clinical severity and long-term outcome of MFS, as well as etiological therapies, are lacking. A comprehensive understanding of both the coding and non-coding transcriptomic landscape, along with epigenetic regulation down to the single-cell level, has contributed significantly to deciphering the underlying molecular pathomechanisms. This has shed light on key members of involved pathways, moving them into researchers’ focus as potential candidates for biomarkers and potential targets for RNA therapy. Several studies on tissue samples from mouse models and MFS patients have identified promising target genes that could prevent the deterioration of aortic aneurysms, such as Tfam, which is crucial for mitochondrial respiration, and miR-632, which is involved in Wnt/β-catenin signaling, End-Mt, and fibrosis, as well as miR-122, associated with the inflammatory response. In contrast, only a very limited number of transcriptomic or epigenetic studies have been conducted on the peripheral blood of MFS patients (Table 1). Among these, miRNA-200c, previously linked to cardiovascular disease, emerged as a biomarker candidate, showing a strong correlation with the status of the aortic root (Z-score). However, further studies applying a multi-omics approach with larger patient cohorts are essential. Investigating rare diseases poses challenges, as access to patient samples is limited, often resulting in underpowered studies. Establishing specialized MFS centers and concentrating clinical and research efforts in a single location could help to overcome this obstacle.

## Figures and Tables

**Table 1:** Genome-Wide Gene Expression and Epigenetic Studies of MFS and non-MFS TAA.

Species	Tissue		Target	Technology	Validation	Study
		Sample Number				
**MFS**						
human	skin fibroblasts	42	mRNA	microarrays	qPCR	[16]
mouse (Fbn1^mgR/mg^)	aortic tissue	3 pools (12 mice)	mRNA	microarrays	qPCR	[19]
mouse (Fbn1^mgR/mg^)	aortic tissue	3 pools	mRNA	RNA-seq	qPCR,	[20]
					functional studies	
mouse (Fbn1^C1039G/+^),	aortic tissue	4	mRNA	RNA-seq	qPCR, functional studies	[28]
human		4				
human	aortic tissue	6	mRNA	RNA-seq	functional studies	[33]
human	blood	7	mRNA,	microarrays	qPCR	[57]
			miRNA			
human	aortic tissue	3	lncRNA	microarrays	qPCR	[59]
human	aortic tissue	8	miRNA	RNA-seq	functional studies	[18]
mouse (Fbn1^mgR/mg^), human	aortic tissue	4	miRNA,	RNA-seq,	qPCR,	[21]
		5	mRNA	microarrays	functional studies	
mouse (Fbn1^C1039G/+^), human	aortic tissue	7	mRNA	scRNA-seq	qPCR,	[84]
		1			functional studies	
human	aortic tissue	3	mRNA	scRNA-seq	functional studies	[85]
mouse (Fbn1^mgR/mg^)	aortic tissue	7	mRNA	scRNA-seq	functional studies	[89]
human	blood	194	methylated DNA	DNA-methylation array	none	[102]
mouse (Fbn1^C1041G/+^)	SMCs	8	mRNA	scRNA-seq,	functional studies	[103]
				ATAC-seq		
**vEDS**						
mouse (Col3a1^G938D/+^),	aortic tissue	6	miRNA	RNA-seq	functional studies	[116]
(Col3a1^G209S/+^)						
human	skin fibroblast	18	miRNA	microarrays	qPCR	[123]
**LDS**						
human	blood	30	lncRNA	Bioinformatics	qPCR,	[127]
					functional studies	
**BAV and MFS**						
human	aortic tissue	48	RNA, Chromatin	RT-PCR, Chromatin Immunoprecipitation	functional studies	[128]
**BAV and TAV**						
human	aortic tissue	32	methylated DNA,	DNA-methylation array,	none	[129]
			mRNA	RNA-seq		
